# Effects of intermittent hypoxia with thrombin in an in vitro model of human brain endothelial cells and their impact on PAR-1/PAR-3 cleavage

**DOI:** 10.1038/s41598-022-15592-x

**Published:** 2022-07-19

**Authors:** Cindy Zolotoff, Clémentine Puech, Frédéric Roche, Nathalie Perek

**Affiliations:** 1 INSERM, U1059, Sainbiose, Dysfonction Vasculaire et Hémostase, Université Jean Monnet Saint-Etienne, Saint-Priest-en-Jarez, France; 2 Service de Physiologie Clinique Et de L’Exercice, Centre VISAS, CHU Saint Etienne, Saint-Priest-en-Jarez, France; 3 Faculté de Médecine - Campus Santé Innovations, 10 Rue de la Marandière, 42270 Saint-Priest-en-Jarez, France

**Keywords:** Brain injuries, Sleep disorders, Cell biology

## Abstract

Patients with obstructive sleep apnea/hypopnea (OSA) are at high risk of cerebrovascular diseases leading to cognitive impairment. The oxidative stress generated by intermittent hypoxia (IH) could lead to an increase in blood–brain barrier (BBB) permeability, an essential interface for the protection of the brain. Moreover, in patients with OSA, blood coagulation could be increased leading to cardiovascular complications. Thrombin is a factor found increased in these populations that exerts various cellular effects through activation of protease activated receptors (PARs). Thus, we have evaluated in an in vitro BBB model the association of IH with thrombin at two concentrations. We measured the apparent BBB permeability, expression of tight junctions, ROS production, HIF-1α expression, and cleavage of PAR-1/PAR-3. Pre-treatment with dabigatran was performed. IH and higher thrombin concentrations altered BBB permeability: high levels of HIF-1α expression, ROS and PAR-1 activation compared to PAR-3 in such conditions. Conversely, lower concentration of thrombin associated with IH appear to have a protective effect on BBB with a significant cleavage of PAR-3. Dabigatran reversed the deleterious effect of thrombin at high concentrations but also suppressed the beneficial effect of low dose thrombin. Therefore, thrombin and PARs represent novel attractive targets to prevent BBB opening in OSA.

## Introduction

Obstructive sleep apnea/hypopnea (OSA) is a disorder associated with repeated intermittent hypoxia-reoxygenation stress during sleep^1^. Chronic intermittent hypoxia (IH) has numerous metabolic and physiological consequences of neurological^2,3^ and cardiovascular^4,5^ types linked to dysfunction of endothelial cells. In fact, IH processes that characterize OSA contribute to an increased risk of cognitive deficit caused by blood–brain barrier (BBB) disruption, allowing access to certain "compounds" from the blood flow to the brain compartment^6^. The BBB is a barrier that isolates the brain from harmful substances that may be in the bloodstream but allows important brain nutrients to pass through^7^. This neuro-vascular unit consists of brain endothelial cells, astrocytes, pericytes and neurons^8^. Endothelial cells are connected by tight junctions (TJs) (zonula occludens (ZO)-1, claudin-5, etc.)^9^ and adherent basal junctions (e.g., vascular endothelial cadherin (VE-cadherin))^10^ that create a high strength paracellular barrier to limit permeability. This brain endothelial cell dysfunction has been shown in apneic patients and animals exposed to intermittent hypoxic cycles^6^. One mechanism that may explain these complications is the oxidative stress generated by IH^2^. Oxidative stress is defined as an imbalance between pro-oxidant and antioxidant systems leading to the production of reactive oxygen species (ROS) and thus the oxidation of biological molecules^11^. IH as well as ROS are then able to modulate different transcription factors such as hypoxia-inducible factor 1α (HIF-1α)^12^. This transcription factor is a marker of a decrease in oxygen. In addition, patients with sleep apnea have an increase of HIF-1 α protein in their sera^13^. Sleep apnea is also known to increase cardiovascular risks characterized by coagulation abnormalities^14,15^. Several factors are increased: hematocrit^16^, blood viscosity^17^, platelet aggregation and activity^18,19^ or coagulation factors including thrombin^20^ which leads to a pro-coagulant state in such disorders.

Thrombin is a key serine protease in the coagulation cascade, allowing the formation of insoluble clots by conversion of fibrinogen to fibrin^21^. This protease is involved in blood clotting and it also interacts with cellular receptors to induce signaling pathways that mediate inflammatory responses. Thrombin receptors are protease activated receptors (PARs). PARs are a family of G protein-coupled receptors, consisting of four members (PAR-1, PAR-2, PAR-3 and PAR-4) and involved in different cellular mechanisms^22^. We were particularly interested in PAR-1 and PAR-3, which have high affinity domains for thrombin recruitment, allowing them to be cleaved efficiently^23^. Whereas, PAR-2 receptor is not cleaved by thrombin and the PAR-4 receptor requires a high concentration of thrombin to be cleaved but can be activated at lower concentrations when its present a heterodimer with PAR-1 or PAR-3. Several studies have demonstrated the role of thrombin and the upregulation of PARs (in particular PAR-1) in the progression of neurodegenerative diseases^24^. Studies have also shown that high concentrations of thrombin promote the occurrence of neurodegenerative disorders. However, low levels of thrombin may allow brain cells to survive various stresses^25–27^. In several in vitro brain endothelial cells, high levels of thrombin may cause a disruption of endothelial cells in line with expression of inflammatory proteins TNFα, IL-6, and MMPs 2 and 9^28^ or with a ROS generation and thrombin receptor PAR-1 cleavage^29,30^. Thrombin activates PAR-1 which enhances HIF-1α, ROS production, and decreases ZO-1 expression leading to BBB opening. Hence, PAR-1 appeared as an important mediator of the effects of thrombin in the brain^24^. On the contrary, low levels of thrombin prevent endothelial cell disruption^31^.

A pathway to elucidate the cellular mechanisms involved in OSA on brain endothelial cell dysfunction is the development of a relevant in vitro model. In particularly, the crosstalk between brain endothelial cells and the interaction of several factors like thrombin. Our objective was to evaluate the effect of thrombin associated or not with IH on the integrity of a brain endothelial cell model. Two conditions of thrombin exposure were evaluated: one condition that prevented brain endothelial cell disruption and a second condition that led to increased permeability.

In an in vitro model of human brain endothelial cells developed in our laboratory, we investigated the effect of IH and hypercoagulability on BBB permeability, two hallmarks found in OSA. To test this hypothesis, we performed cycles of IH protocol as we had previously done in our laboratory^32^. Three concentrations of thrombin were used (1,5 or 10 U/mL), for 1 h alone or associated with IH in the last hour^31^ to mimic hypercoagulability found in patients after IH and thus evaluate the effect of several concentrations of thrombin on endothelial cells. BBB permeability was performed with TEER and apparent permeability (Papp) measurements, as well as studying the expression of VE-cadherin, claudin-5 and ZO-1 junction proteins. Then we evaluated the level of intracellular ROS and the expression of HIF-1α and PAR-1 and PAR-3 cleavage. Finally, we used a direct thrombin inhibitor (dabigatran) to investigate whether this anticoagulant could limit the adverse effects of thrombin in our model. Indeed, in some studies the use of dabigatran decrease the expression of inflammatory and oxidative stress proteins, implying that thrombin may play a central role in endothelial injury^28^.

The aims were the following (1) to evaluate the effect of thrombin alone and thrombin associated with IH on the integrity of a BBB model; (2) to study the effects of thrombin and IH on thrombin receptors PAR-1 and PAR-3; (3) to study the reversal of thrombin effect with an anticoagulant strategy on brain endothelial cell permeability: ROS, HIF-1α and thrombin receptors in order to predict the possible protective effects.

## Results

### Impact of IH and thrombin on endothelial cell integrity

#### Opposite effects on TEER and Papp measurements with two concentrations of thrombin

**Figure 1:**
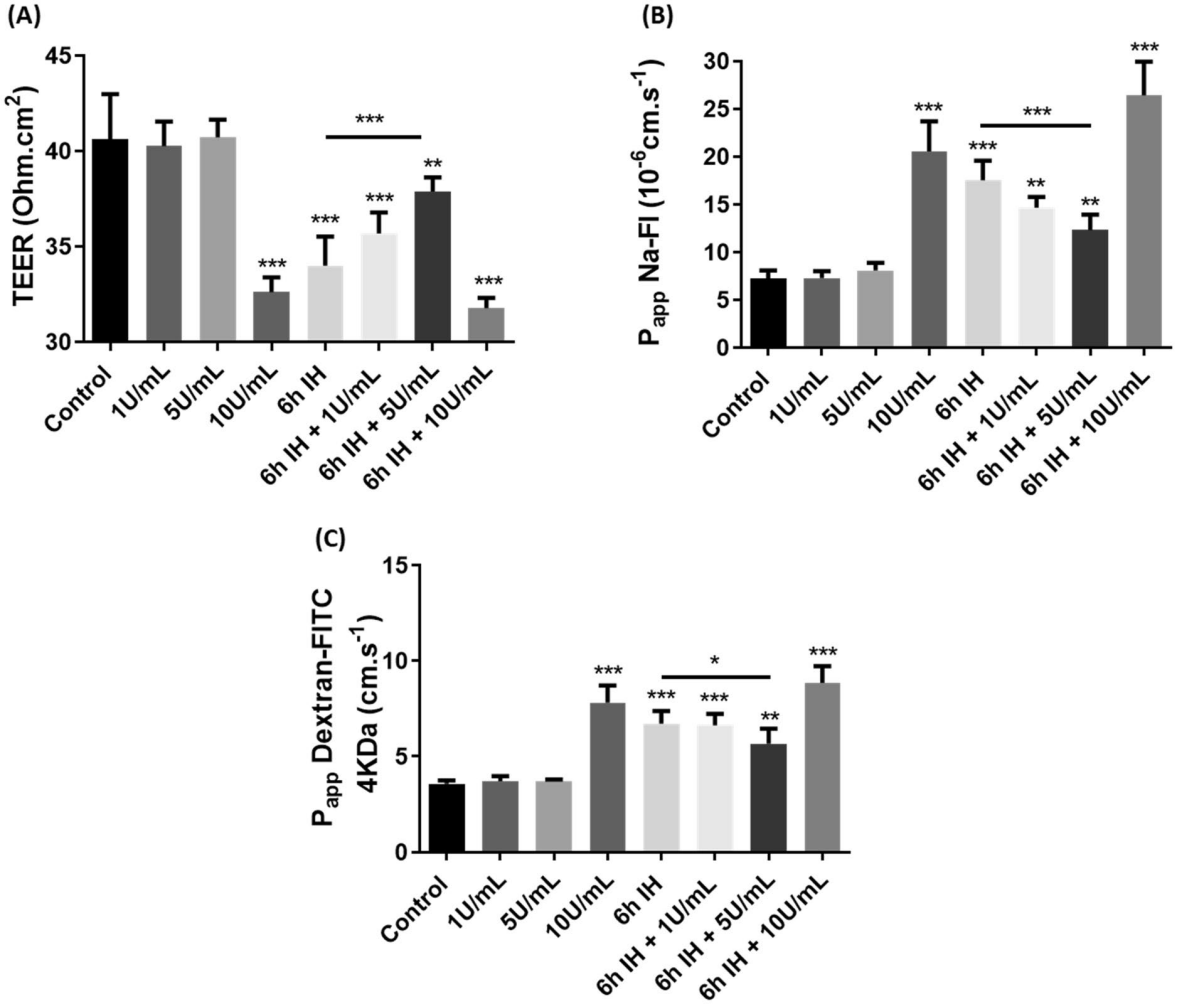
Transendothelial electrical resistance (TEER) (**a**) and apparent permeability (P_app_) for Na–Fl (**b**) or FITC-dextran (**c**) in our different conditions. For treatment with thrombin, cells were exposed to thrombin at 1 or 5 or 10 U/mL for 1 h. Then cells were also exposed to 6 h of IH alone or associated with thrombin at 1 or 5 or 10 U/mL during the last hour. Results are represented as mean value ± SD (*n* = 9, *N* = 3), ** *p* ≤ 0.01, ***p ≤ 0.001 compared to control condition.

As a first step, we looked at the effect of different concentrations of thrombin (1 or 5 or 10 U/mL) for 1 h and the association with IH for 6 h with thrombin (1 or 5 or 10 U/mL) in the last hour in our in vitro model of BBB. To do this, we first investigated the impact on TEER (Fig. 1a). At 1 U/mL and 5 U/mL we did not observe any significant difference with control. TEER decreased significantly at 10 U/mL, 6 h of IH, and 6 h of IH associated with 10 U/mL of thrombin with values varying from 40.6 ± 2.4 to 32.6 ± 0.8 Ω·cm^2^, 34 ± 1.5 Ω·cm^2^, 32 ± 0.5 Ω·cm^2^, respectively (*p* < 0.001). When 6 h of IH was associated with 1 U/mL of thrombin in the last hour we observed a lower decrease of TEER with values from 40.6 ± 2.4 to 35.7 ± 1 Ω.cm^2^ (*p* < 0.01) and even less significant when IH was associated with 5 U/mL of thrombin with values from 40.6 ± 2.4 to 37.9 ± 0.7 Ω.cm^2^ (*p* < 0.01).

Then, Papp with Na-Fl (Fig. 1b) or dextran-FITC (Fig. 1c) were measured in our different conditions. At 1 U/mL and 5 U/mL we did not observe any significant differences compared to control with these two fluorescent molecules. We observed a significant increase at 10 U/mL, 6 h of IH and 6 h of IH associated with 10 U/mL of thrombin with values varying from 7.3 × 10–6 ± 0.8 × 10–6 to 20 × 10–6 ± 3.1 × 10–6 cm.s-1, 17 × 10–6 ± 2 × 10–6 -cm·s-1 and to 26 × 10–6 ± 3.5 × 10–6 cm·s-1 (*p* < 0.001), respectively, for Na-Fl. With dextran-FITC, significant increase was also observed with Papp values varying from 3.6 × 10–6 ± 0.2 × 10–6 to 7.8 × 10–6 ± 0.9 × 10–6 cm.s-1 ,6.7 × 10–6 ± 0.6 × 10–7 cm.s-1 and to 8.8 × 10–6 ± 0.9 × 10–7 cm.s-1 (*p* < 0.001) for 10 U/mL, 6 h of IH, and 6 h of IH associated with 10 U/mL, respectively. When 6 h of IH was associated with 1U/mL of thrombin, we observed a lower increase with a Papp value of 14 × 10–6 ± 1 × 10–6 cm.s-1 for Na-Fl and 6.6 × 10–6 ± 0.6 × 10–6 for dextran-FITC and even less significant when IH was associated with 5 U/mL of thrombin with a Papp value of 12 × 10–6 ± 1.6 × 10–6 cm.s-1 for Na-Fl and 5.6 × 10–6 ± 0.8 × 10–6 for dextran-FITC (*p* < 0.01).

Thus, we observed that thrombin seemed to have different effects depending on its concentration. Indeed, at 1 U/mL it already seems to limit the effects of IH but at 5 U/mL the effect was even more significant on IH. On the contrary, at 10 U/mL of thrombin we observe a deleterious effect on our model. Thus, for the rest of our experiments we used thrombin at 5 U/mL and at 10 U/mL, two concentrations which seem to have an opposite effect on IH.

#### Protective effects of 5U/mL of thrombin on intermittent hypoxia on the expression of protein junctions

After treatment with thrombin, IH and IH associated with thrombin we also evaluated the expression of tight and adherent junction proteins with whole cell ELISA. We evaluated the expression of claudin-5, ZO-1 (TJ proteins) and VE-cadherin (adherent junction protein) since they were involved in IH as well as thrombin effects on endothelial cells^33,34^. With 5 U/mL of thrombin, no significant differences were observed in the expression of junction proteins.

**Figure 2:**
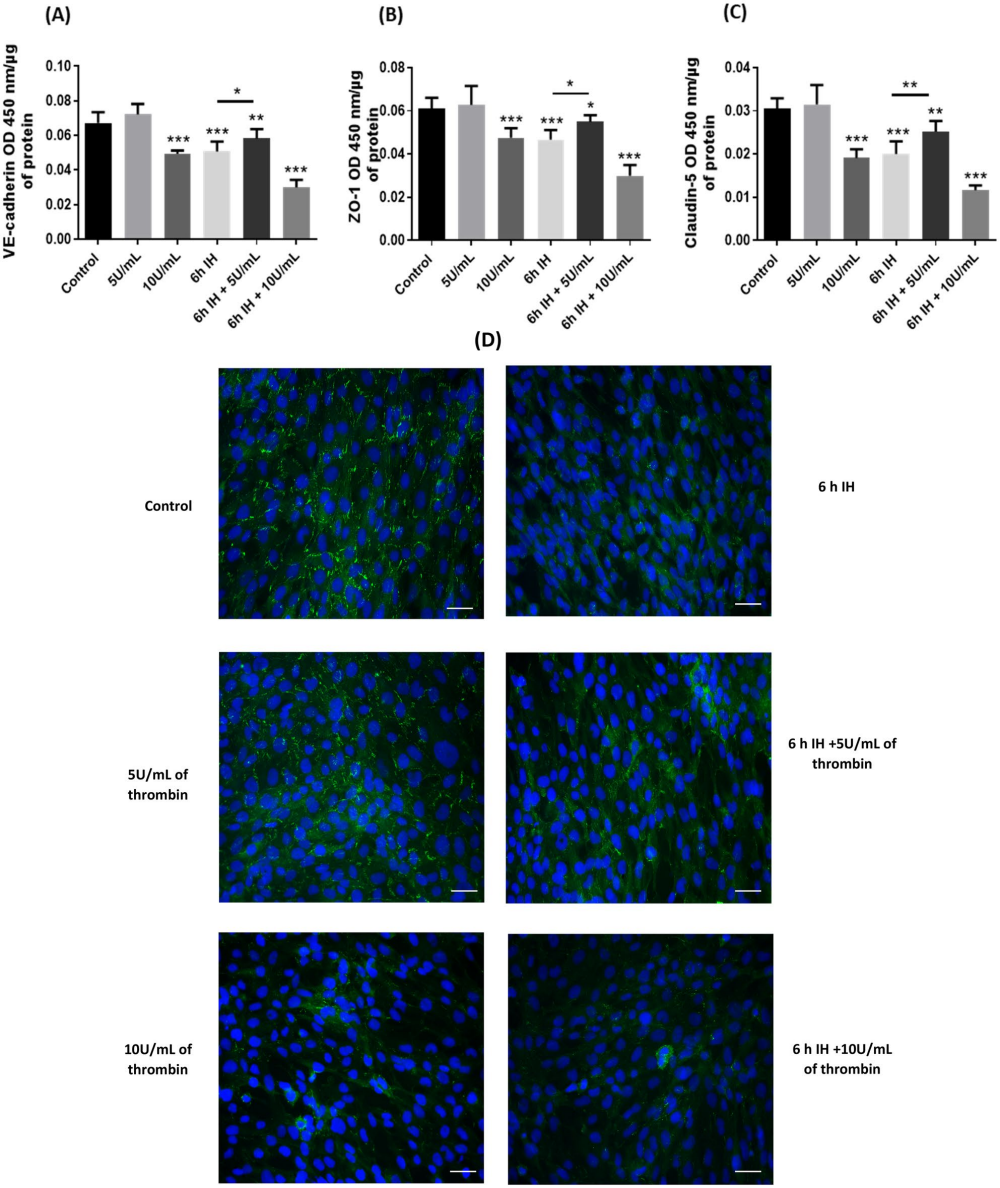
Expressions of VE-cadherin (**a**), ZO-1 (**b**) and claudin-5 (**c**) after exposure of cells in our different conditions. For treatment with thrombin, cells were exposed to thrombin at 5 or 10 U/mL for 1 h. Then cells were also exposed to 6 h of IH alone or associated with thrombin at 5 or 10 U/mL during the last hour. Immunostaining of ZO-1 after exposure of cells to our different treatments in our model of blood–brain barrier (**d**) Scale bar = 100 µm. Results are represented as mean value ± SD (*n* = 9, *N* = 3), *p ≤ 0.05, **p ≤ 0.01, ***p ≤ 0.001 compared to control conditions.

However, with 10 U/mL of thrombin, 6 h of IH and 6 h of IH associated with 10 U/mL of thrombin, we observed a significant decrease in the expression of all proteins (VE-cadherin, ZO-1 and claudin-5). Expression of VE-cadherin (Fig. 2a) significantly decreased by 26%, 25%, 55%, respectively (*p* < 0.017). For TJs, expression of ZO-1 (Fig. 2b) also significantly decreased by 24%, 25% and 53% (*p* < 0.045). Claudin-5 expression (Fig. 2c) showed a decrease of 36%, 33% and 63% for thrombin at 10 U/mL, 6 h of IH, and 6 h of IH associated with 10 U/mL of thrombin, respectively. When 5 U/mL of thrombin is associated with IH, there was a lower decrease in the expression of these junction proteins. We observed a decrease of 15% for VE-cadherin, 11% for ZO-1, and 13% for Claudin-5.

In order to illustrate the whole cell ELISA, we realized IF assays on the ZO-1 TJ protein (Fig. 2d), a marker largely used to observe brain endothelial cell disruption. IF ZO-1 assays confirmed whole cells assays; BBB showed important disruptions at cell–cell junction zones at 6 h of IH, 10 U/mL of thrombin, and when IH was associated with thrombin at 10 U/mL compared to control. However, thrombin at 5 U/mL did not show any significant difference to the control.

### Lower expression of HIF-1α and production of ROS with thrombin at 5U/mL associated with IH

**Figure 3:**
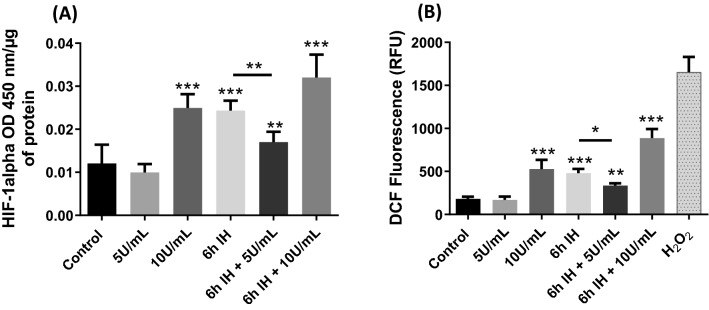
Expressions of HIF-1α (**a**) and ROS production (**b**) in our different conditions. For treatment with thrombin, cells were exposed to thrombin at 5 or 10 U/mL for 1 h. Then cells were also exposed to 6 h of IH alone or associated with thrombin at 5 or 10 U/mL during the last hour. Values are presented as mean value ± SD (*n* = 9, *N* = 3), * *p* ≤ 0.05, **p ≤ 0.01, ***p ≤ 0.001 compared to control condition. IH: intermittent hypoxia, ROS: reactive oxygen species, HIF-1α: hypoxia-inducible factor 1 alpha, DCF: dichlorodihydrofluorescein.

We therefore analyzed the expression of the transcription factor HIF-1 α, a central regulator of hypoxia (Fig. 3a). HIF-1α has been shown to be also stimulated after thrombin exposure^35^.

Thrombin at 5 U/mL did not show a significant difference with control. However, with 10 U/mL of thrombin for 1 h, 6 h of IH or 6 h of IH combined with 10 U/mL of thrombin there was a significant increase in the expression of this factor of 107%, 100% and 165%, respectively. Nevertheless, in the 6 h of IH combination associated with 1 h of thrombin at 5 U/mL, there was a significant increase in HIF-1α expression (41%) but less significant than under the previous conditions.

Next, we observed the generation of ROS after IH and/or thrombin exposure, detectable through the DCFH-DA molecule which, once oxidized by the ROS, will allow the formation of DCF (visible by fluorescence) (Fig. 3b). In the same way as for HIF-1α, we did not observe any difference between 5 U/mL thrombin for 1 h in our model with the control. However, an increase in fluorescence intensity was visible under the same conditions as HIF-1α. For 10 U/mL of thrombin for 1 h, ROS generation showed an important threefold increase from 181 ± 24 to 528.1 ± 105 relative fluorescence units (RFU) (*p* < 0.001). Under IH conditions, ROS production showed a threefold increase from 181 ± 24 to 478.4 ± 51 RFU (*p* < 0.001). For the association between 6 h of IH and 10 U/mL of thrombin, ROS production showed a fivefold increase from 181 ± 24 to 886.9 ± 105 RFU (*p* < 0.001). Finally, with the combination of IH and 5U/mL of thrombin, a much lower generation of ROS was observed with values from 181 ± 24 to 335.4 ± 26 RFU (*p* = 0.0050).

### Highest expression of PAR-1 with 10U/ml of thrombin and PAR-3 with 5U/mL of thrombin

**Figure 4:**
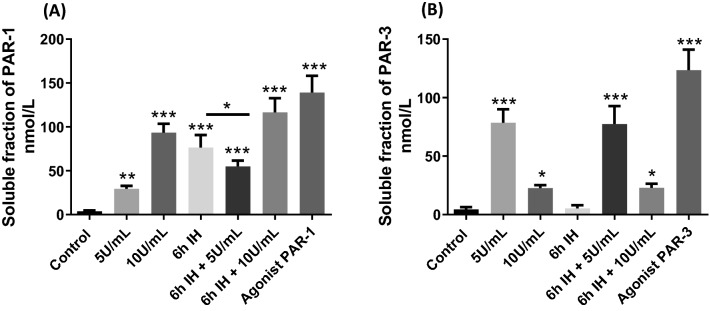
Evaluation of the expression of the soluble fraction of PAR-1 (**a**) and PAR-3 (**b**) in the supernatant of HBEC-5i after different treatments. For treatment with thrombin, cells were exposed to thrombin at 5 or 10 U/mL for 1 h. Then cells were also exposed to 6 h of IH alone or associated with thrombin at 5 or 10 U/mL during the last hour. Values are presented as mean value ± SD (*n* = 6, *N* = 3), * *p* ≤ 0.05, **p ≤ 0.01, ***p ≤ 0.001 compared to control condition. IH: intermittent hypoxia, PAR: protease activated receptor.

We then evaluated the release of the soluble fractions of PAR1 and PAR-3; since to be activated PARs must be cleaved (Fig. 4). The cleaved form indicates that PARs were active which is the reason we evaluated only the soluble fraction. These receptors, once cleaved, lead to a multitude of responses, which may explain the effects observed in our model.

The lowest concentration of thrombin (5 U/mL) allowed a cleavage of PAR-1 to 29.4 ± 3.4 nmol/L (*p* = 0.0022) soluble fraction of PAR-1; and this value was increased approximately 3 times with the highest concentration of thrombin to obtain a high cleavage value of 93.5 ± 10.1 nmol/L (*p* < 0.001) soluble fraction of PAR-1 (Fig. 4a). Under conditions of IH and IH combined with thrombin at 10 U/mL there was also significant cleavage of PAR-1 respectively of 76.4 ± 14.4 nmol/L and 116.6 ± 16.2 nmol/L (*p* < 0.001) soluble fraction of PAR-1. However, when IH was combined with thrombin at a concentration of 5 U/mL, a lower soluble fraction of PAR-1 was observed of 55 ± 6.7 nmol/L (*p* < 0.001).

For PAR-3 the opposite was observed (Fig. 4b). The highest concentration of thrombin (10 U/mL) allowed a cleavage of PAR-3 to 22.5 ± 2.4 nmol/L (*p* = 0.0476) soluble fraction of PAR-3; and this value was increased approximately 4 times with the lower concentration of thrombin to obtain a high cleavage value of 78.6 ± 11.5 nmol/L (*p* < 0.001) soluble fraction of PAR-3. Under conditions of 6 h of IH, no significant difference was observed with control. 6 h of IH associated with thrombin at 10 U/mL allowed a slight cleavage of PAR-3 (probably due to the presence of 10 U/mL of thrombin) of 23 ± 3.5 nmol/L (*p* = 0.0430).

However, when IH was combined with thrombin at a concentration of 5 U/mL, a soluble fraction of PAR-3 similar to 5 U/mL of thrombin alone was observed of 77.4 ± 15.4 nmol/L (*p* < 0.001). (e).

### Impact of PAR-1 and PAR-3 agonists on endothelial cell integrity

**Figure 5:**
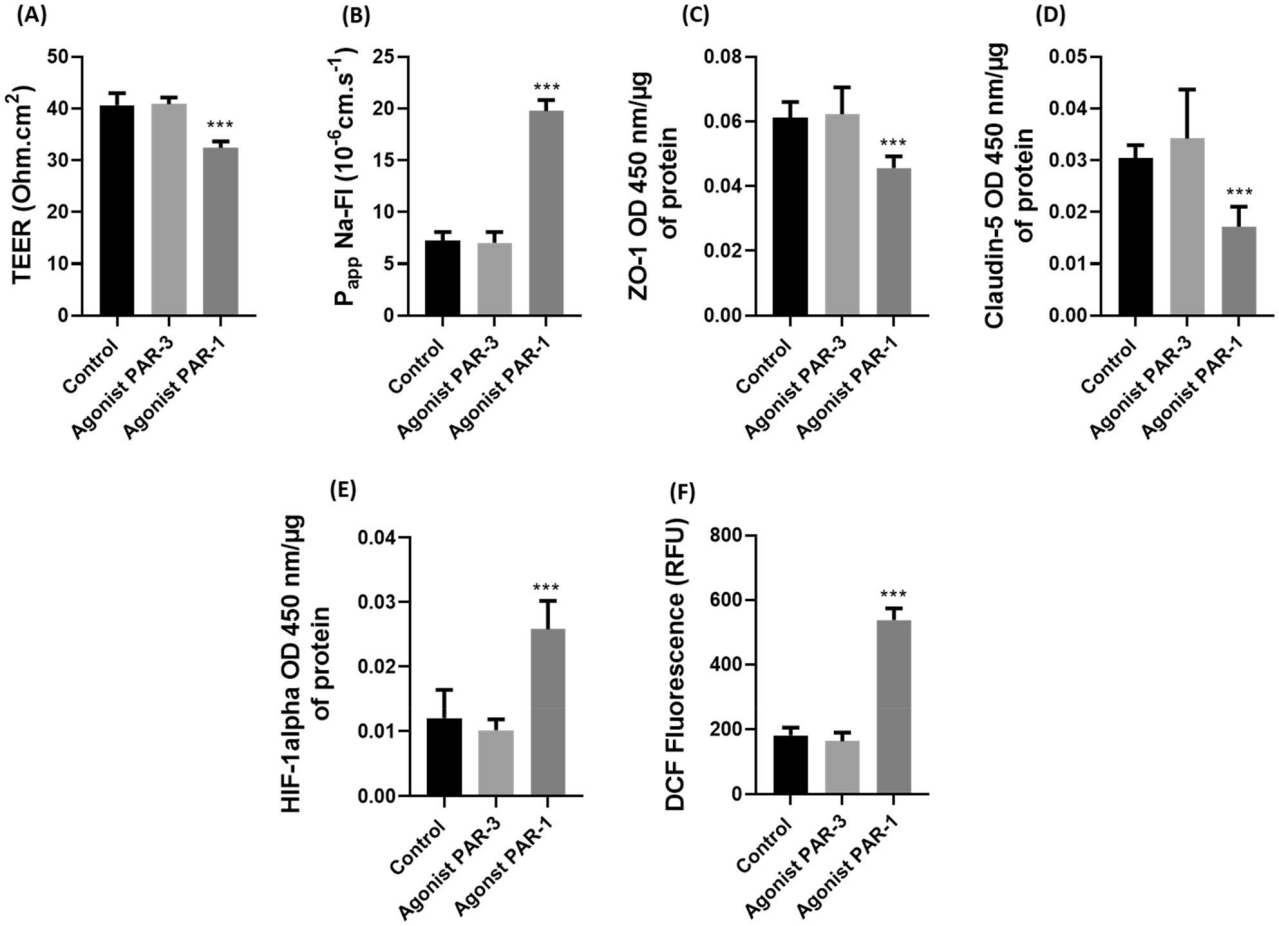
Evaluation of transendothelial electrical resistance (TEER) (**a**)**,** apparent permeability (P_app_) for Na-Fl (**b**), expressions of ZO-1 (**c**), Claudin-5 (**d**), HIF-1α (**e**) and ROS production (**f**) with PAR-1 and PAR-3 agonists. Values are presented as mean value ± SD (*n* = 9, *N* = 3), ***p ≤ 0.001 compared to control condition. PAR: protease activated receptor.

After evaluating the expression of soluble PAR-1 and PAR-3 factors (Fig. 4), we thus decided to investigate the effects of these agonists on our in vitro BBB model (Fig. 5). Initially we investigated the impact on TEER (Fig. 5a) and Papp with Na-Fl (Fig. 5b). We observe a significant decrease in TEER with PAR-1 agonist with values varying from 40.6 ± 2.4 to 32.4 ± 1.2 Ω.cm^2^ and a significant increase in Papp with values varying from 7.3 × 10–6 ± 0.8 × 10–6 to 19.8 × 10–6 ± 1 × 10–6 cm.s-1 for Na-Fl (*p* < 0.001). The PAR-3 agonist did not show any significant difference compared to the control in both experiments.

Then, we evaluated the expression of ZO-1 and claudin-5 with these agonists (Fig. 5c and d). we observed a significant decrease in the expression of these proteins with the PAR-1 agonist. Indeed, the expression of ZO-1 (Fig. 5c) significantly decreased by 26% (*p* < 0.001) and by 49% for Claudin-5 (Fig. 5d) (*p* = 0.0002). We did not observe a significant difference with the PAR-3 agonist.

We therefore analysed the expression of the transcription factor HIF-1α (Fig. 5e) and the generation of ROS (Fig. 5f) with agonists PAR-1 and PAR-3. There was a significant increase in HIF-1α expression (110%) with PAR-1 agonist. ROS generation showed an important threefold increase from 181 ± 24 to 537.1 ± 36 relative fluorescence units (RFU) (*p* < 0.001) with PAR-1 agonist. Here also PAR-3 agonist did not show a significant difference with control.

### Pretreatment with dabigatran suppresses the effects of thrombin

#### Impact on endothelial cell integrity: effects of intermittent hypoxia

**Figure 6:**
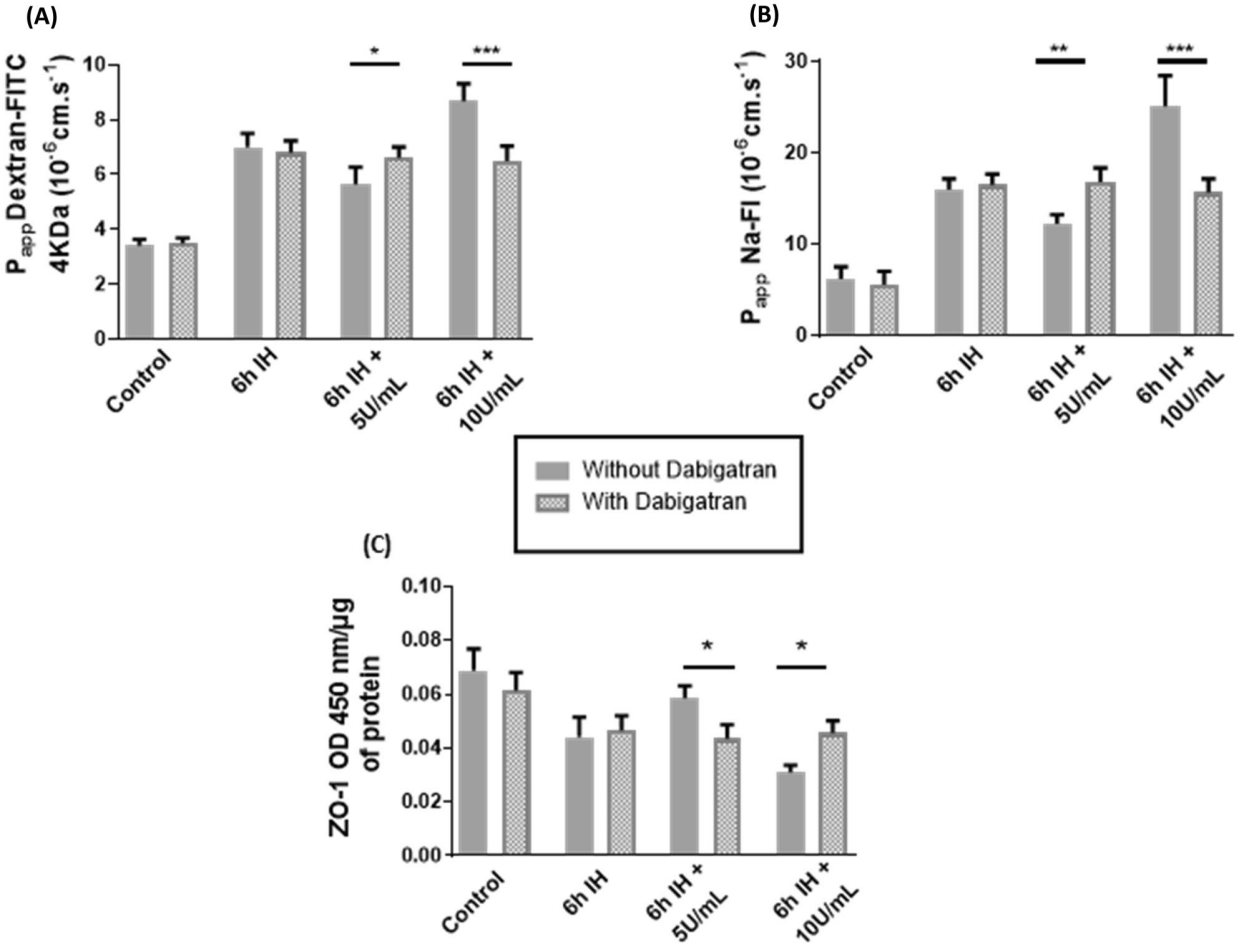
Apparent permeability (Papp) for FITC-dextran (**a**) or Na–Fl (**b**) and expressions of ZO-1 (**c**) after pretreatment with dabigatran in our cycles of IH with or without thrombin at 5U/mL and 10U/mL. Results are represented as mean value ± SD (*n* = 9, *N* = 3), *p ≤ 0.05, **p ≤ 0.01, ***p ≤ 0.001. *IH* intermittent hypoxia.

Finally, we proposed to use dabigatran, a direct thrombin inhibitor, to evaluate a preventive role it could have on thrombin associated with IH on the BBB. We evaluated the pretreatment of this anticoagulant (at 0.5 µM pharmacological concentrationq based on Hawkins^36^ for 24 h) on the endothelial monolayer with IH alone and IH associated with both thrombin concentrations in the last hour (Fig. 6).

We observed that pretreatment with dabigatran suppressed the beneficial effects of thrombin at 5 U/mL but protects against the deleterious effects of thrombin at 10 U/mL. Indeed, with dabigatran at 6 h of IH associated with 5 U/mL of thrombin, we observed a P_app_ value of 6.6 × 10^–6^ ± 3.9 × 10^–7^ cm.s^-1^ (*p* = 0.0286) and a P_app_ value of 16.8 × 10^–6^ ± 1.5 × 10^–6^ cm.s^-1^ (*p* = 0.012), respectively, for dextran and Na-Fl similar to the P_app_ values observed in IH conditions (Fig. 6a and b). Pretreatment with dabigatran on 6 h of IH associated with 10 U/mL decreased permeability, with a P_app_ value of 6.5 × 10^–6^ ± 5.4 × 10^–7^ cm.s^-1^ (*p* < 0.001) for dextran and 15.7 × 10^–6^ ± 1.5 × 10^–6 -^cm.s^-1^ (*p* < 0.001) for Na-Fl (Fig. 6a and b).

Dabigatran suppressed the positive effect of 5 U/mL of thrombin on ZO-1 expression. However, dabigatran prevented the decrease of ZO-1 expression with thrombin at 10 U/mL in whole cell ELISA assay (Fig. 6c), probably related to the non activation of PAR-1.

#### Dabigatran suppresses PARs activation by thrombin

**Figure 7:**
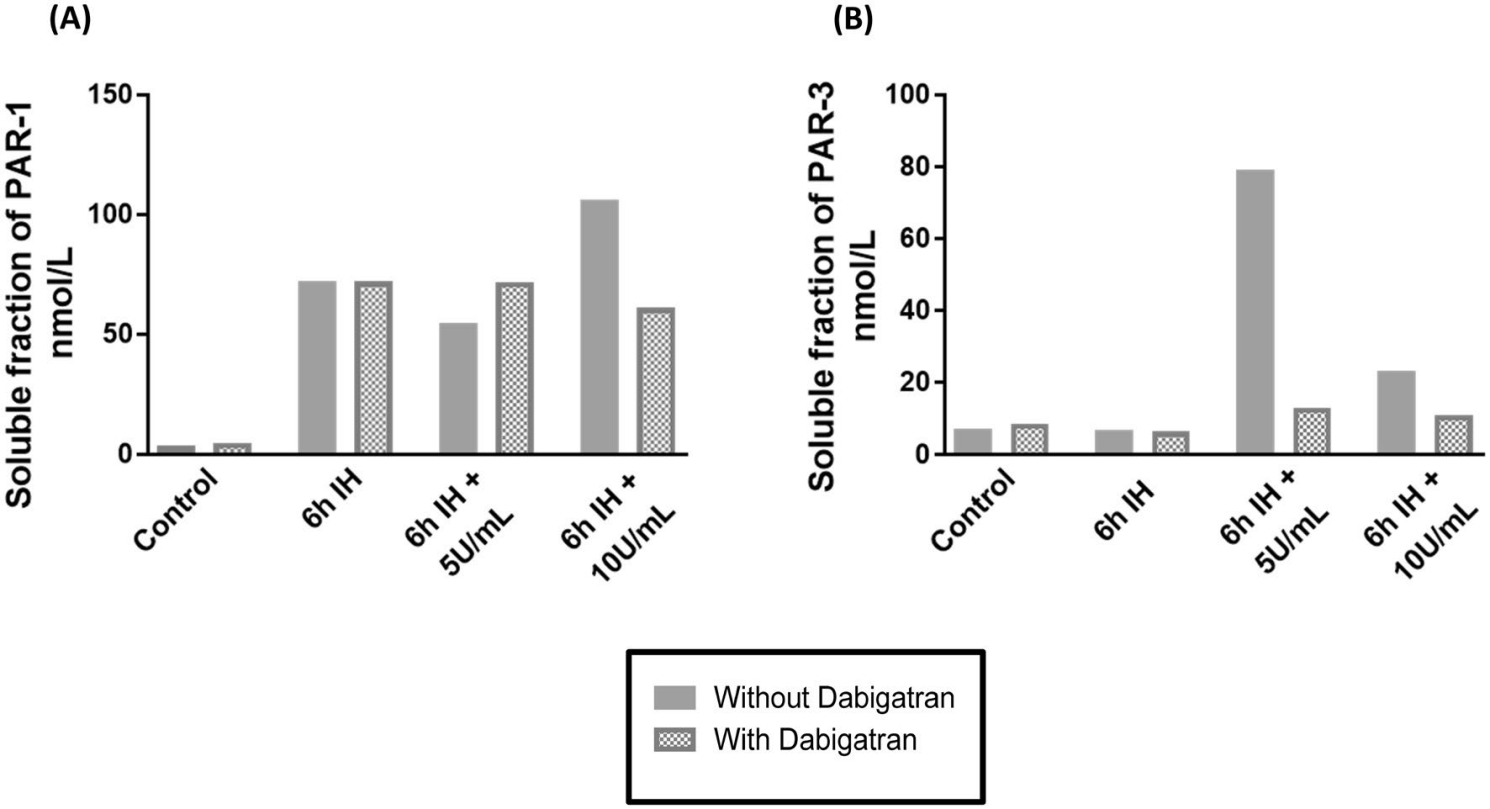
Evaluation of the expression of the soluble fraction of PAR-1 (**A**) and PAR-3 (**B**) in the supernatant of HBEC-5i after pretreatment with dabigatran in our cycles of IH with or without thrombin at 5U/mL and 10U/mL (*n* = 6, *N* = 2). *IH* intermittent hypoxia, PAR: protease activated receptor.

Next, we wanted to evaluate the impact of this thrombin inhibition with dabigatran on PAR-1 and PAR-3 and more specifically on their cleaved parts (Fig. 7).

For PAR-1, greater cleavage was observed when inhibiting thrombin at 5U/mL (Fig. 7a); lower cleavage was observed when IH was associated with 5 U/mL of thrombin (Fig. 7b). These results are in line with higher PAR-1 activation on increasing P_app_ and a decrease in ZO-1, without beneficial effect of PAR-3. We suppressed the protecting role.

For PAR-1 and for PAR-3, less cleavage was observed when inhibiting thrombin at 10 U/mL (Fig. 7a and b).

## Discussion

OSA is a risk factor for vascular disease^37^ mediated by pathophysiological cascades, including the increase of procoagulant factors^15^. These factors include thrombin^20,38^, a coagulation factor that is indispensable for the production of blood clots but also known to influence the occurrence of neurodegenerative diseases at high concentrations^24^.

Several hypotheses raise the possibility that OSA characterized by IH may be responsible for the alteration of the BBB and thus responsible for subsequent cognitive disorders^6^. However, thrombin is also known to alter BBB functionality in vitro at high concentrations^29,30,34^. There is increasing evidence that thrombin is expressed locally in the central nervous system. Its role in brain injury depends on its concentration, as higher amounts cause neuroinflammation and apoptosis, while lower concentrations might even have a cytoprotective effect^39^. Nevertheless, the BBB is an efficient interface for the protection of the central nervous system, allowing an exchange between the bloodstream and the brain through TJs and transporters^40^. To date, no studies combine IH and the presence of clotting factor to evaluate the mediated effects on the BBB. Therefore, it seemed evident to us to compare the effect of thrombin alone at different concentrations, IH cycles and finally IH cycles associated with thrombin in an in vitro model of the human BBB.

Initially, we studied the permeability of the BBB as well as the expression of the BBB junctional proteins. In our first study^32^, our IH cycles revealed an alteration of the BBB through an increase in membrane permeability and a decrease in the expression of protein junctions (Figs. 1 and 2). However, the thrombin concentrations applied to our model of the BBB had opposite effects already seen by others^24^. Indeed, 5 U/mL of thrombin for 1 h, did not alter the integrity of the BBB. On the contrary, with 10 U/mL of thrombin, we observed an alteration of the BBB, which has already been demonstrated^29,34^. Therefore, the combination of 6 h of IH and 10 U/mL of thrombin could only further alter the BBB, which was found in our study and was marked by an even greater membrane permeability (Fig. 1). These were associated with a very significant decrease in protein junction expression (Fig. 2). However, the combination of 5 U/mL of thrombin with 6 h of IH showed a rather beneficial effect on the BBB, since membrane permeability compared to IH cycles alone was significantly decreased and the expression of junction proteins was more important (Fig. 1 and 2).

Then we determined which factors could explain the alteration of the BBB in some of our conditions (Fig. 3). Oxidative stress generated by IH and thrombin at high concentration has been shown to increase ROS levels^41,42^. An increase in these ROS then disturbs homeostasis and is known to alter the BBB through an alteration of the TJs^43–45^. In correlation with the increase in ROS, an increase in the transcription factor HIF-1α, a marker of hypoxia^12,46^, was also observed. Numerous studies have suggested that activation of HIF-1α-mediated signaling disrupts TJs, resulting in increased BBB permeability^47,48^. In our study, we found a production of ROS and an important expression of HIF-1α under our conditions where the junction proteins are the most decreased and where the permeability is the greatest (10 U/mL of thrombin, 6 h of IH, 6 h of IH associated with 10 U/mL of thrombin). Conversely, with 5 U/mL of combined thrombin in the last hour of the IH cycles, ROS production and HIF-1α expression were lower, suggesting that different pathways are established.

We were interested to see what pathways could explain the expression of these factors and the relative protective effect observed with 5 U/mL of thrombin. We already know that thrombin acts via PARs. PARs are a unique class of G protein-coupled transmembrane receptors. They have recently been identified for their involvement in intestinal permeability under physiological and pathological conditions^49^ but are also known to affect vascular responses.

There are four such receptors, of which the most studied is PAR-1, but PAR-3 is also cleaved by thrombin^22^. Both have high affinity domains allowing them to be cleaved efficiently by thrombin^23,50^. Afterwards, several signaling cascades are activated (such as the phosphatidylinositol 3-kinase (PI3-K) pathway, the mitogen-activated protein kinase cascade or the Rho kinase pathway) leading to different cellular responses^51^, which may explain our results. Thus, we investigated the cleavage of PAR-1 and PAR-3, allowing us to see how these receptors are activated (Fig. 4).

Thrombin at both concentrations (5 and 10 U/mL) cleaved PAR-1, but a higher cleavage was observed with 10 U/mL of thrombin (Fig. 4A). Numerous studies interested in neurodegenerative diseases found an increased cleavage and expression of PAR-1 at high concentrations^25,52^. Surprisingly, a significant cleavage of PAR-1 has also been found during cycles of IH, such as in an in vitro mammary cancer cell study^53^. Thus, the combination of IH and thrombin at 10 U/mL, due to their additive effects, significantly increased the cleavage of PAR-1. We observed that it is under conditions where PAR-1 cleavage is important that we have an important alteration in our in vitro model of the BBB, with increased ROS production and significant HIF-1α expression. Furthermore, the use of the PAR-1 agonist alone in our model shows an alteration of endothelial cell integrity marked by an increase in its permeability associated with a decrease in the expression of junctional proteins (Fig. 5). Indeed, several studies have also shown that during PAR-1 cleavage, the production of ROS as well as the expression of HIF-1α are increased^29,35^ and could therefore be responsible for the alteration of our model of BBB permeability. Furthermore, the cleavage of PAR-1 with 5U/mL of thrombin associated with IH was less than with IH alone, suggesting the involvement of an alternative pathway.

Then we studied the cleavage of the PAR-3 receptor, which could explain this protective effect of such a combination on the barrier. Indeed, a study showed that a low concentration thrombin had a protective signaling activity on endothelial cells: when the cells were treated with siRNA for the PAR-3, the protective effect was eliminated^54^. In our study, a significant increase in cleavage of PAR-3 was observed with the lowest dose of thrombin (5 U/mL). At 10 U/mL of thrombin we had a very low cleavage. Furthermore, hypoxia exposure alone did not allow cleavage of PAR-3 (Fig. 4B). PAR-3 agonist was not related to HIF-1α and ROS and prevented deleterious effects of PAR-1 on ZO-1, Claudin-5 and on apparent permeability (Fig. 5).

Finally, we used dabigatran, a selective thrombin inhibitor. Dabigatran as chronic therapy reduces the recurrence of venous thromboembolism and cardioembolic stroke^55^ which can be increased in OSA and in particular by an increased level of thrombin^56^. It has been approved for the prevention of stroke and blood clots associated with nonvalvular atrial fibrillation^57^. Our experiments with dabigatran clearly demonstrated the loss of the protective effect of thrombin at 5 U/mL when inhibited (Figs. 6 and 7). Indeed, permeability increased, TJs were further altered with increased PAR-1 cleavage and no significant PAR-3 cleavage suggesting that not only PAR-1 but also PAR-3 release influence on cellular response depending on the level of thrombin stimulation.

**Figure 8:**
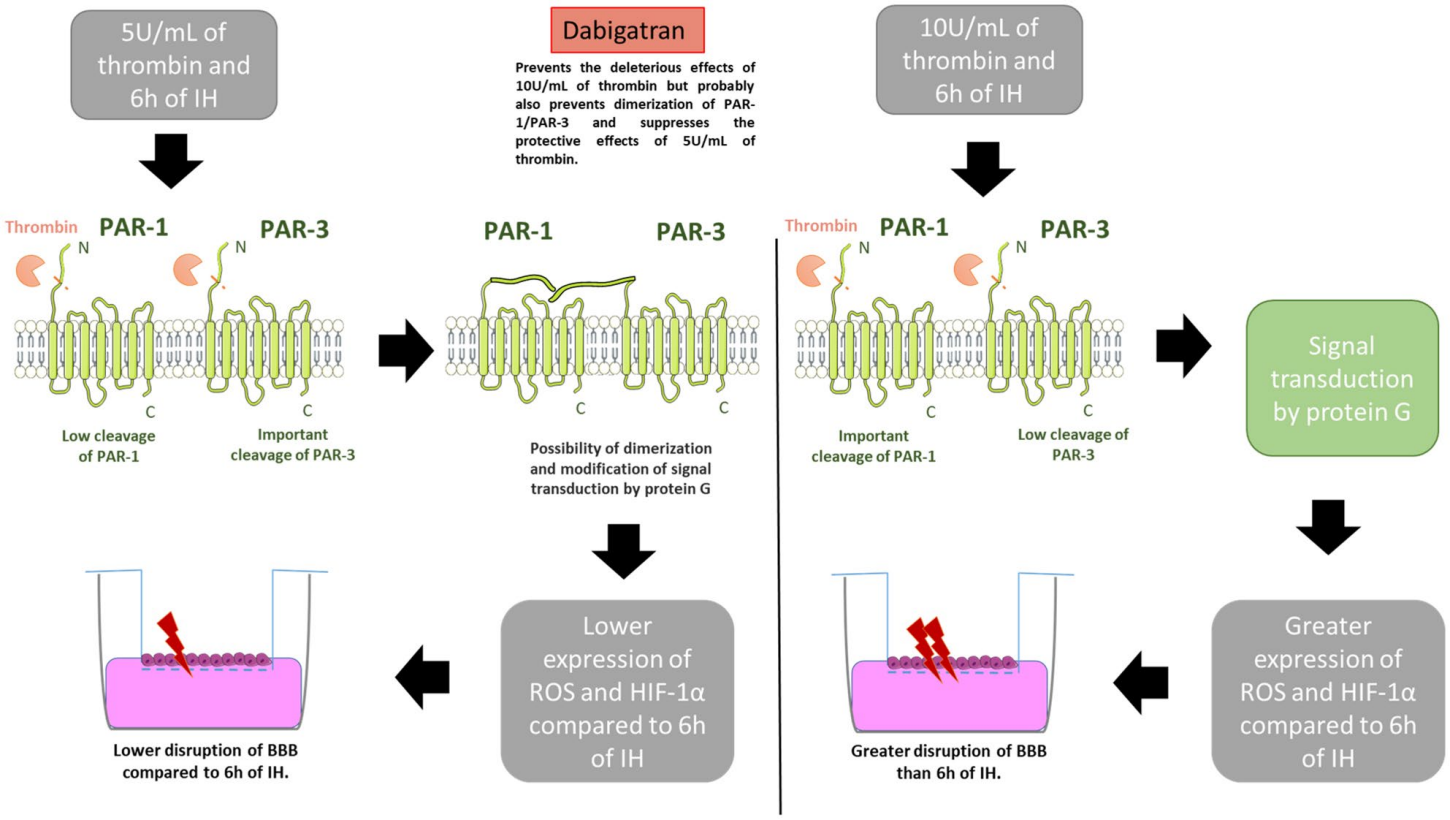
One hypothesis that may explain the dual effects of thrombin concentration associated with intermittent hypoxia on our in vitro model of the blood brain-barrier (BBB). HIF-1α: hypoxia-inducible factor 1 α; IH: intermittent hypoxia; PAR-1: protease activated receptor 1; PAR-3: protease activated receptor 3, ROS: reactive oxygen species.

The mechanisms that would explain the protective response of a low dose of thrombin are still unknown. However, in view of our results, several hypotheses can explain the thrombin concentration-dependent effects. One is that there are two populations of PAR-1 with high and low affinity on the surface of endothelial cells. Low concentrations of thrombin would preferentially activate the receptors leading to a protective response. This protective response could in particular be mediated by the PI3-K pathway since in a study using a specific PI3-K inhibitor, the protective signaling responses by low concentrations of thrombin were blocked^31^. Another possibility suggested that a low concentration of thrombin could be mediated by activation of two receptors, PAR-1 and PAR-3^31^. Here, we clearly demonstrated in the present study the activation of both receptors and preferentially PAR-3 during low thrombin concentration, which seems to modify the initial signaling cascade. Indeed, a study showed that PAR-1 could form heterodimers with PAR-3 and that dimerization could then modulate the G-protein coupling specificity of PAR-1 in endothelial cells^58^ (Fig. 8). PAR-3 would then be involved in the regulation of PAR-1 signaling, which in turn regulates the endothelial permeability by different mechanisms^58^.

There are several limitations in our studies such as thrombin concentrations which reflect a first approach of the mechanisms that may be involved. Moreover, there could be a difference with results observed in vivo. Thus, it would be interesting to study the direct impact of IH cycles in an in vivo rodent model in order to provide measurements in serum of different coagulation factors such as thrombin during IH exposure. In addition, because of the close role between thrombin, platelets and PARs (PAR-1 and PAR-4)^59^ it would be interesting to include platelets in our model. Especially since in patients with OSA, we find an increase in platelet activity^18,60^ as well as platelet aggregation^19^. Moreover, based on numerous studies, these overactivated platelets as well as the high expression of various factors during aggregation appear to influence the permeability of the BBB^61–63^.

## Conclusion

To summarize, our model of chronic IH associated with thrombin in an in vitro model of human BBB allowed us to represent an interesting first approach in sleep apnea patients with a suspected procoagulant state. Thrombin concentration is thought to play a major role in the intracellular responses mediated by PARs leading to neurodegenerative disorders. Indeed, it appears that low concentrations of thrombin (5 U/mL) promote PAR-3 activation and bring protective signaling for brain endothelial cells, marked by a decrease in ROS formation and HIF-1α expression. Conversely, at higher concentrations (10 U/mL of thrombin), PAR-1 is believed to be further cleaved and responsible for a signaling cascade that alters the integrity of the BBB. Chronic IH generated by our cycles also leads to significant cleavage of PAR-1. Thus, further studies are required to understand the exact cellular signaling mechanisms that occur and their impact in sleep apnea and blood hypercoagulation.

## Materials and methods

### Chemicals and reagents

Rabbit polyclonal to VE-cadherin (VE-cadherin-ab33168, RRID:AB_870662) for ELISA was from Abcam (Cambridge, United Kingdom). Rabbit polyclonal anti-ZO-1 (ZO-1-ab59720, RRID: AB_946249) for immunofluorescence (IF) was from Abcam (Cambridge, United Kingdom). Mouse monoclonal anti-claudin-5 (sc-374221, RRID:AB_10988234) and secondary antibody for cell ELISA mIgG BP-HRP (sc-516102, RRID:AB_2916301) were from Santa Cruz Biotechnology (Dallas, TX, USA).

Rabbit ZO-1 polyclonal antibody for ELISA (40-2200, RRID:AB_2533456), Dulbecco's Modified Eagle Medium (DMEM)/Nutrient Mixture F-12 (11330057), rhodamine-123, secondary antibodies for IF: goat anti-rabbit antibodies (A-11034 Alexa Fluor 488, RRID:AB_2576217), DAPI for microscopy, superFrost microscope slide and Coomassie Plus protein assay were obtained from Thermo Fisher Scientific (Waltham, USA). Vectashield Antifade mounting medium (H-1000) was from Eurobio Scientific.

Cell culture inserts for 24-well plates (high density pore, 0.4-μm pore diameter size, translucent polyterephthalate ethylene membrane, culture flasks and companion plates were from Dominique Dutscher (Strasbourg, France). Imaging plates FC, 96-well, TC-surface were from MoBiTec (Goettingen, Germany).

Astrocyte medium, astrocyte growth supplement, fetal bovine serum and penicillin–streptomycin solution for astrocytes were from CliniSciences (Nanterre, France). Amphotericin B (30–003-CF) and trypsin were from Corning (Manassas, USA). Penicillin–streptomycin (100X) was from PanReac Applichem (Darmstadt, Germany).

Human PAR-1 and PAR-3 kits (MBS733919, MBS7269065) were from COGER (Paris, France). Dabigatran was obtained from Alsachim (Illkirch, France). PAR-3 agonist was from Eurogentec (Angers, France).

OxiSelect Intracellular ROS Assay Kit (green fluorescence, STA-342) and HIF- 1α Cell Based ELISA Kit (CBA-281) were from Cell Biolabs (San Diego, USA).

All other reagents were from Sigma-Aldrich (St Quentin Fallavier, France).

### In vitro BBB model

The BBB model was composed of HBEC-5i endothelial cells (from ATCC-Manassas, VA, USA) cultivated with human astrocytes (HA) cell line (from ScienCell Research Laboratories, Carlsbad, CA, USA) on transwells with 0.4-µm pores and in plates.

Initially, HBEC-5i were cultured in DMEM/F12 in contact with astrocytic conditioned medium. The conditioned medium corresponds then to a medium that has remained in contact with the astrocytes for 48 h. HBEC-5i with HA conditioned medium reached an optimal steady state plateau at day 14 which was maintained for 5 d. Cells were cultured at 37 °C with 5% CO_2_^64^.

### Treatments

For cycles of IH, we used a hypoxic workstation: Baker Ruskinn, Invivo_2_ 300 and the ICO_2_N_2_IC Advanced Gas Mixing System (Maine, USA). Under IH, cells were exposed to repeated hypoxia (35 min, 1% O_2_)/reoxygenation (25 min, 18% O_2_) for 6 cycles^32^. For treatment with thrombin, cells were exposed to thrombin at 1 or 5 or 10 U/mL for 1 h in order to mimic blood coagulation and to observe the impact of different concentrations of thrombin^36^.

Then cells were also exposed to 6 h of IH associated with thrombin at 1 or 5 or 10 U/mL in the last hour.1U/mL of thrombin corresponds to 10 nM.

Cells were incubated with dabigatran (inhibitor of thrombin) at 0.5 µM pharmacological concentration^36^. We also used agonists of PAR-1 (sequence: Ser-Phe-Leu-Arg-Asn-Pro-Asn-Asp-Lys-Tyr-Glu-Pro-Phe) and PAR-3 (sequence: H-Ser-Phe-Asn-Gly-Gly-Pro-NH2) pathways.

### Barrier properties

P_app_ was assessed using two hydrophilic fluorescent molecules: sodium fluorescein (Na-Fl MW = 376 Da) and fluorescein isothyocyanate dextran (FITC-dextran: MW = 4 kDa). First, the medium was removed, and cells were washed with Ringer HEPES. After that,10 µg/mL of Na-Fl or FITC-dextran in Ringer HEPES was loaded onto the luminal side of the insert and incubated at 37 °C for 1 h. Fluorescence of Na-Fl and FITC-dextran were measured with a fluorescence spectrophotometer (Fluoroskan Ascent, Thermo Fisher Scientific, France) at excitation of 485-nm and emission of 530-nm wavelengths. P_app_ is expressed in cm.s-^1^ and was calculated using the formula used in our previous work (Puech^34^).

TEER was recorded using an EVOM resistance meter with STX-2 electrodes to characterize the formation of a tight endothelial cell monolayer. One electrode was placed on the luminal side and the other electrode on the abluminal side. TEER measurements of inserts with cells were subtracted by the blank inserts, and the result was multiplied by the membrane surface to obtain the TEER measurement in Ω.cm^2^.

### Whole cell ELISA

Cells were cultivated in permeable 96-well permeable plates at a density of 10,000 cells/well at 37 °C with conditioned medium of human astrocytes. At confluence, cells were exposed to IH, thrombin or IH associated with thrombin (with or without dabigatran).

Firstly, cells were fixed with 4% paraformaldehyde for 20 min and then, permeabilized with methanol with H_2_O_2_ for 20 min at room temperature. Then nonspecific sites were blocked. Cells were incubated with mouse anti-claudin-5 (2 µg/mL), rabbit anti-ZO-1 (5 µg/mL) and VE-cadherin (1/200) at room temperature for 2 h or overnight at 4 °C. Secondary IgG antibody diluted 1/2500 or 1/500 was incubated at room temperature for 2 h. After washing, tetramethylbenzidine (TMB) substrate was added into each well for 10 min in the dark at room temperature. The color reaction was measured at 450-nm with a spectrophotometer and normalized to total amount of proteins by Coomassie blue total protein assay, according to the manufacturer’s instructions.

### Quantification of HIF-1α

For this factor, cells were cultured into permeable 96-well plates with conditioned medium of HA. Different conditions were then performed on these cells (intermittent hypoxia cycles and/or different thrombin concentrations). Then HIF-1α expression was measured with a cell-based ELISA assay (Cell Biolabs) according to the manufacturer’s instructions.

### IF assays

Firstly, cells of inserts were fixed and permeabilized with glacial methanol for 6 min. After a blocking step, rabbit anti-ZO-1 was diluted 1/200 in the blocking solution for 1 h at 37 °C. The secondary antibody was diluted 1/500 in the blocking solution for 45 min at 37 °C. Nuclei were detected using DAPI diluted 1/1000 in the blocking solution for 30 min at room temperature. The inserts were then placed on glass slides and covered with a Vectashield mounting medium, and glass coverslips were placed on the inserts. The junction staining was performed using an epifluorescence microscope (Zeiss, Axioskop 40) equipped with ZEN software.

### Determination of intracellular ROS levels

To determine ROS activity in our model, we used the OxiSelect™ Intracellular ROS Assay Kit. Cells were seeded with conditioned medium of HA in permeable 96-well plates and then incubated with dichlorodihydrofluorescein diacetate (DCFH-DA) at 37 °C for 1 h. Then cells were exposed to thrombin, IH or IH associated with thrombin (with or without dabigatran). After that, fluorescence was read with a fluorimetric plate reader at 480-nm/530-nm. ROS results were determined by comparison with a DCF standard curve.

### Thrombin and PAR-1/ PAR-3 pathway

PAR-1 and PAR-3 were activated by cleavage of their N-terminal domains. Thus, PAR-1 and PAR-3 activation were evaluated with the cleavage of PAR-1 and PAR-3 based on competitive ELISAs. The intensity of the color is inversely proportional to the PAR3 concentration since PAR3 from samples and PAR3-HRP conjugate compete for the anti-PAR3 antibody binding site. Since the number of sites is limited, as more sites are occupied by PAR3 from the sample, fewer sites are left to bind PAR3-HRP conjugate. Measurements were realized according to the manufacturer’s recommendation.

### Statistical analysis

Statistical analysis was performed using Graphpad Prism 6 software (San Diego, CA, USA). These results were tested with one-way ANOVA using Tukey′s post hoc tests. When P-values were < 0.05, the differences between means were considered to be significant and the values are expressed as the mean ± SD.

## Data Availability

The datasets generated for this study are available from the corresponding author upon reasonable request.
